# Oxytocin treatment improves dexamethasone‐induced depression‐like symptoms associated with enhancement of hippocampal CREB‐BDNF signaling in female mice

**DOI:** 10.1002/npr2.12271

**Published:** 2022-06-21

**Authors:** Masayoshi Mori, Hiromi Shizunaga, Hiroyoshi Harada, Yuki Tajiri, Yusuke Murata, Kazuki Terada, Kenji Ohe, Munechika Enjoji

**Affiliations:** ^1^ Department of Pharmacotherapeutics, Faculty of Pharmaceutical Sciences Fukuoka University Fukuoka Japan; ^2^ Division of Pharmacotherapeutics, Faculty of Pharmaceutical Sciences Himeji Dokkyo University Himeji Japan

**Keywords:** depression, female, glucocorticoid, hippocampus, oxytocin

## Abstract

**Aims:**

Chronic stress and glucocorticoid exposure are risk factors for depression. Oxytocin (OT) has been shown to have antistress and antidepressant‐like effects in male rodents. However, depression is twice as common in women than in men, and it remains unclear whether OT exerts antidepressant‐like effects in women with depression. Therefore, in this study, we investigated the therapeutic effect of chronic OT administration in a female mouse model of dexamethasone (DEX)‐induced depression.

**Methods:**

Female C57BL/6J mice were administered saline (vehicle, s.c.), DEX (s.c.), or OT (i.p.) + DEX (s.c.) daily for 8 weeks, and then assessed for anxiety‐ and depression‐like behaviors. We also examined the hippocampal levels of phosphorylated cAMP response element‐binding protein (p‐CREB) and brain‐derived neurotrophic factor (BDNF), which are important mediators of the response to antidepressants.

**Results:**

Simultaneous OT treatment blocked the adverse effects of DEX on emotional behaviors. Furthermore, it upregulated p‐CREB and BDNF in the hippocampus.

**Conclusion:**

OT may exert antidepressant‐like effects by activating hippocampal CREB‐BDNF signaling in a female mouse model of depression.

## 1 INTRODUCTION

Stress, a major risk factor for depression, is among the leading causes of disability worldwide and is twice as common in women than in men.^1^ However, only about half of patients treated with conventional antidepressants achieve remission.^2^ Therefore, it is important to find better antidepressant agents. The hippocampus modulates mood and the hypothalamic–pituitary–adrenal (HPA) axis, which is one of the endocrinological mechanisms against stress stimuli.^3^, ^4^ Hippocampal dysfunction and dysregulation of the HPA axis, including elevated plasma levels of cortisol in humans and corticosterone (CORT) in rodents, are implicated in the pathogenesis of depression.^5^, ^6^, ^7^ The cAMP response element‐binding protein (CREB) and its transcriptional targets, such as brain‐derived neurotrophic factor (BDNF), play important roles in regulating neuronal growth and neuroplasticity of the hippocampus.^8^ Thus, modulation of the HPA axis and hippocampal CREB‐BDNF signaling may be beneficial for the treatment for depression.^9^, ^10^, ^11^

Oxytocin (OT) is mainly produced in the paraventricular nucleus (PVN) of the hypothalamus. OT neurons in the PVN project to the hippocampus.^12^ OT has been shown to act as a buffer against stress by reducing HPA axis activity and enhancing hippocampal synaptic plasticity.^13^, ^14^, ^15^ Based on these findings, OT has potential as a new treatment for depression.^16^ However, the tendency to only use male subjects for experiments is a major limitation,^17^ which is restricting in studies investigating the antidepressant‐like effect of OT, because women are at a greater risk of depression than men.^1^ In rodent models, chronic exposure to glucocorticoids, such as dexamethasone (DEX), reflects chronic stress load conditions. This strategy is widely used to induce the depression phenotype and to evaluate the efficacy of antidepressant drugs.^18^ Thus, here, we assess the therapeutic effects of chronic OT administration in a female mouse model of DEX‐induced depression.

## 2 METHODS

### 2.1 Animals

Twenty‐four female C57BL/6J mice (age: 10 weeks; Charles River Laboratories) were used in this study. They were individually housed under a 12‐:12‐h light–dark cycle, with lights turned on at 07:00 h, and free access to food and water.

### 2.2 Drug administration

OT (4 016 373, Bachem, Switzerland) and DEX (2392‐39‐4; Fujifilm Wako Pure Chemical Co., Osaka, Japan) were dissolved in saline. Mice received saline (s.c.), DEX (5 mg/kg, s.c.), or DEX and OT (1 mg/kg, i.p.) daily for 56 consecutive days. The dosages of OT and DEX were determined per previous studies.^19^, ^20^

### 2.3 Experimental design

**FIGURE 1:**
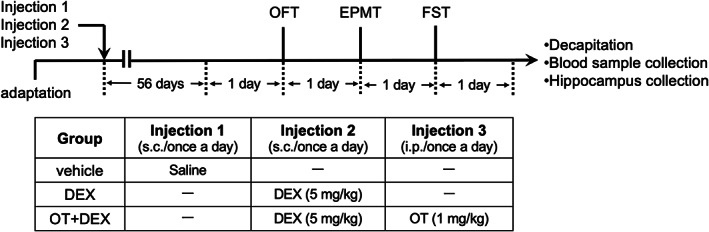
Experimental design. All mice were administered with the vehicle (saline, S.c.), DEX (5 mg/kg, s.c.), or OT (1 mg/kg, I.p.) + DEX (5 mg/kg, s.c.) for 8 weeks. After the termination of drug administration, the mice were subjected to OFT, EPMT, and FST every other day. The animals were then decapitated 24 h after the FST for the evaluation of plasma CORT levels and hippocampal CREB‐BDNF signaling. BDNF, brain‐derived neurotrophic factor; CORT, corticosterone; CREB, cAMP response element‐binding protein; DEX, dexamethasone; EPMT, elevated plus maze test; FST, forced swimming test; OFT, open field test; OT, oxytocin

The animals were acclimated for 1 week after arrival and then randomly assigned to the following experimental groups (*n* = 8 per group): (a) vehicle group, treated with saline; (b) DEX group, treated with DEX; and (c) OT + DEX group, treated with OT and DEX ( Figure 1 ). After termination of drug administration, all animals were subjected to a series of behavioral tests. Then, they were euthanized under anesthesia with sodium pentobarbital (50 mg/kg), and blood samples and dorsal and ventral hippocampi were collected.

### 2.4 Open field test (OFT)

The OFT was conducted using an apparatus consisting of a circular arena (diameter: 60 cm) with a surrounding wall (height: 50 cm). Mice were individually placed in the center of the arena and behavior was recorded for 5 min using a video camera positioned above the arena. The total distance traveled by the mice within the arena—indicated locomotor activity—was measured using video‐tracking software (SMART, Panlab Harvard Apparatus, Barcelona, Spain).

### 2.5 Elevated plus maze test (EPMT)

The EPMT was performed using a platform consisting of two closed arms (6 × 31.5 cm) with 15‐cm‐high walls and two open arms of the same size with no walls. The height of the platform was 40 cm above the room floor, and all four arms were connected to a square zone (6 × 6 cm) with no walls. Animals were placed in the central area facing one open arm and allowed to explore the maze for 5 min. Fewer open arm entries indicated anxiety‐like behavior.

### 2.6 Forced swimming test (FST)

Each mouse was forced to swim for 6 min in a glass cylinder (diameter: 14 cm; height: 20 cm), containing water filled to a depth of 12 cm at a 25 ± 1°C. The duration of immobility of each animal was recorded for the last 4 min. Increased duration of immobility indicated depression‐like behavior.

### 2.7 Plasma CORT level

Trunk blood samples were collected into tubes containing 5 μl heparin. Then, the samples were centrifuged at 830 *g* for 15 min at 4°C to separate the plasma, which was assayed using a commercially available CORT ELISA kit per the manufacturer's instructions (EC3001‐1, AssayPro, St. Charles, MO, USA).

### 2.8 Western blotting

The following primary antibodies were used overnight at 4°C: anti‐BDNF (1:1000, ab108319; Abcam, Cambridge, UK), anti‐phosphorylated‐CREB (p‐CREB; 1:1000, 9198; Cell Signaling Technology, Beverly, MA, USA), anti‐CREB (1:1000, 9197; Cell Signaling Technology), and anti‐β‐actin antibodies (1:10 000, 4967; Cell Signaling Technology). The membranes were washed and incubated with a secondary antibody (1:5000, 7074; Cell Signaling Technology) for 1 h at 20–25°C. Immunoreactive bands were visualized with an enhanced chemiluminescence reagent and quantified using ImageJ (NIH, Bethesda, Maryland, USA).

### 2.9 Statistical analysis

Data were analyzed using StatView v5 (HULINKS, Tokyo, Japan). Group comparisons were performed using parametric tests such as one‐way analysis of variance (ANOVA), followed by Bonferroni/Dunn post hoc analysis, when required. All data are represented as mean ± standard error of the mean. Statistical significance was set at *P* < 0.05.

## 3 RESULTS

**FIGURE 2:**
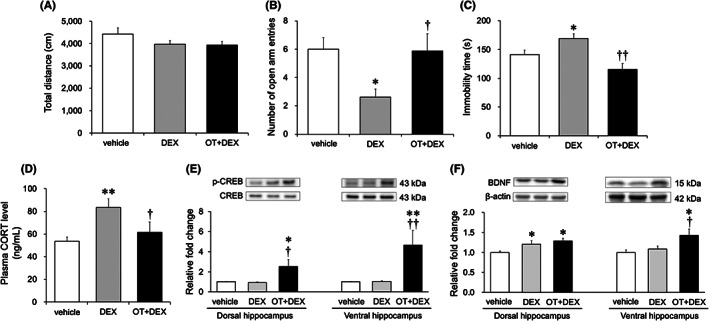
Effects of chronic OT and DEX administration on symptomatic characteristics of depression. Chronic OT and DEX administration did not affect locomotor activity in the OFT (A). Chronic DEX administration significantly decreased anxiety‐like behavior and the number of open arm entries in the EPMT, which were prevented by cotreatment with OT (B). Chronic DEX administration significantly increased depression‐like behavior and duration of immobility in the FST, which were reversed by the cotreatment with OT (C). The DEX group showed significantly higher HPA axis activity and plasma CORT levels, which were prevented by cotreatment with OT (D). The OT + DEX group showed significantly higher phosphorylated‐CREB protein expression levels in both the dorsal and ventral hippocampus; however, no significant difference was found between the saline and DEX groups (E). High expression levels of BDNF in the dorsal hippocampus were observed in the DEX and OT + DEX groups compared to those in the saline group (F, *left‐hand side*). The OT + DEX group showed significantly higher BDNF protein expression levels in the ventral hippocampus; however, no significant difference was found between the saline and DEX groups (F, *right‐hand side*). The number of animals per group is 8. Bars represent mean ± standard error of the mean. Statistical analysis was performed by one‐way ANOVA followed by Bonferroni/Dunn post hoc tests. *P < 0.05, **P < 0.01 vs. saline group, †*P <* 0.05, ††*P <* 0.01 vs. DEX group. DEX, dexamethasone; EPMT, elevated plus maze test; FST, forced swimming test; HPA, hypothalamic–pituitary–adrenal; CORT, corticosterone; OFT, open field test; OT, oxytocin

The effects of chronic OT and DEX administration on emotional behaviors and plasma CORT levels in female mice are shown in Figure 2A–D. Although there were no significant changes in locomotor activity in the OFT among the groups (Figure 2A), one‐way ANOVA indicated significant differences in the EPMT (*F*_(2,21)_ = 4.433, *P =* 0.0248), FST (*F*_(2,21)_ = 9.299, *P =* 0.0013), and plasma CORT levels (*F*_(2,21)_ = 4.741, *P =* 0.0200). Subsequent post hoc analysis revealed a lower number of open arm entries in the DEX group than in the vehicle group (*P* < 0.05) and OT + DEX group (*P* < 0.05) in the EPMT (Figure 2B). In the FST, the immobility duration of the DEX group was longer than that of the vehicle group (*P* < 0.05) and OT + DEX group (*P* < 0.01) (Figure 2C). Similarly, the DEX group showed higher plasma CORT levels than the vehicle group (*P* < 0.01) and OT + DEX group (*P* < 0.05) (Figure 2D).

Figure 1E,F depict the effects of chronic OT and DEX administration on hippocampal p‐CREB and BDNF protein expression levels. One‐way ANOVA indicated a significant intergroup difference in the p‐CREB levels in both the dorsal (*F*_(2,21)_ = 4.433, *P =* 0.0173) and ventral hippocampus (*F*_(2,21)_ = 4.433, *P =* 0.0075). The OT + DEX group showed a higher p‐CREB level in the dorsal hippocampus than those shown by the vehicle group (*P* < 0.05) and DEX group (*P* < 0.05) (Figure 2E). Similarly, p‐CREB levels in the ventral region were higher in the OT + DEX group than in the vehicle group (*P* < 0.01) and DEX group (*P* < 0.01) (Figure 2E). Furthermore, one‐way ANOVA revealed a significant difference in BDNF levels in both the dorsal (*F*_(2,21)_ = 4.278, *P =* 0.0276) and ventral hippocampus (*F*_(2,21)_ = 4.251, *P =* 0.0282) among the groups. The OT + DEX and DEX groups showed higher BDNF levels in the dorsal hippocampus than the vehicle group (*P* < 0.05 and *P* < 0.05, respectively) (Figure 2F). The OT + DEX group showed higher BDNF levels in the ventral hippocampus than the vehicle and DEX groups (*P* < 0.05 and *P* < 0.05, respectively) (Figure 2F).

## 4 DISCUSSION

Herein, the therapeutic effects of chronic OT administration in a female mouse model of DEX‐induced depression were investigated. DEX exposure increased anxiety‐ and depression‐like behaviors and plasma CORT levels, while simultaneous OT treatment blocked the adverse effects of DEX, and upregulated p‐CREB and BDNF levels in the dorsal and ventral hippocampus. Our results suggest that OT exerts antidepressant‐like effects in a female mouse model of depression.

Dysregulation of the HPA axis is often observed in patients with depression and anxiety.^5^ This dysregulation can be improved with chronic antidepressant treatment and serves as a biological marker for depression.^10^ Furthermore, a recent study showed that female mice chronically treated with DEX exhibited an increase in the serum levels of CORT.^21^ OT is related to psychiatric disorders such as depression and anxiety disorder.^15^, ^16^ In this study, we found that chronic OT treatment counteracted the adverse effects of DEX by reducing plasma CORT levels and depression‐ and anxiety‐like behaviors without altering locomotor activity. These results are consistent with previous findings on the antidepressant and anxiolytic effects of OT in a male animal model of depression.^16^, ^20^, ^22^

The hippocampus is functionally segregated along the dorsolateral/ventromedial axis. The dorsal region is involved in diverse cognitive functions, while the ventral region is linked to emotion and stress response, including negative feedback regulation of the HPA axis.^3^, ^4^ Previous studies have shown that hippocampal CREB‐BDNF signaling is important in the maintenance of hippocampal function and is inhibited by chronic stress and glucocorticoid exposure, particularly in the ventral subregion, which is implicated in depression.^7^, ^23^ Long‐term antidepressant treatment activates CREB‐BDNF signaling and subsequently promotes hippocampal functions such as neurogenesis.^6^, ^9^, ^11^ Similarly, OT mediates antistress and antidepressant‐like effects by enhancing hippocampal neuronal plasticity. For instance, OT protects against stress‐induced decreases in hippocampal neurons by acting on OT receptors in male rodents.^13^, ^14^ Furthermore, OT ameliorates anxiety‐ and depression‐like behaviors by increasing BDNF expression levels and neurogenesis in the hippocampus in a male rodent model of depression.^22^ These observations suggest that the antidepressant effects of OT might be at least partly mediated by activation of CREB‐BDNF signaling in the hippocampus,^9^, ^24^ which is in line with our results.

Chronic glucocorticoid treatment affects the hippocampal CREB‐BDNF signaling pathway in male mice;^18^ however, in this study, DEX‐treated female mice had an increased level of BDNF that was not accompanied by an activation of p‐CREB expression in the dorsal hippocampus. Although the precise mechanisms are unknown, several chemical treatments affecting BDNF expression levels in the hippocampus without altering the p‐CREB activity have been reported.^25^, ^26^ Furthermore, male rodents are more vulnerable to glucocorticoids than females rodents.^27^ Thus, the sex‐dependent effects of DEX may be associated with the results in this study. Additionally, there are sex differences in the effects of OT on behavior and brain activation in both humans and animals.^28^ OT treatment increased social behavior and the activity of OT neurons in the hypothalamus in male rats, but not female rats.^29^ Furthermore, female rodents have lower OT receptor binding densities in the hippocampus compared with males rodents.^30^ Thus, future studies will need to compare the antidepressant‐like effects of OT on DEX‐induced model animals of depression between sexes.

Overall, this study is the first to show that chronic OT treatment exerts antidepressant‐like effects in a female mouse model of DEX‐induced depression. Additionally, our results indicate that enhancement of hippocampal CREB‐BDNF signaling by OT, preferentially in the ventral part, may normalize the impairment of emotional behaviors and HPA axis activity induced by DEX. Our findings highlight the importance of OT as a potential treatment to manage depression in women.

## AUTHORS' CONTRIBUTIONS

M.M. designed the experiments. M.M., H.S., H.H., and Y.T. performed the experiments and analyzed the results. M.M. and K.T. wrote the manuscript. Y.M. and K.O. drafted the manuscript. M.E. supervised all the aspects of this study.

## CONFLICT OF INTEREST

None.

## INFORMED CONSENT

None.

## REGISTRY AND REGISTRATION NO. OF THE STUDY/TRIAL

None.

## ANIMAL STUDIES

Animal experiments were approved by the University of Fukuoka Committee on Animal Research (approval number: 1813105; January 29, 2019).

## Supporting information


Data S1

## Data Availability

The raw data are available as supplementary material (Data S1).
